# Real-time CT/MR fusion imaging-guided core needle biopsy: enhancing diagnostic precision in head and neck lesions

**DOI:** 10.1186/s13244-026-02426-x

**Published:** 2026-10-09

**Authors:** Xing Liang, Ying Wang, Zheng Yuan, Jiliang Ren, Gongxin Yang, Weiqing Tang, Ying Yuan

**Affiliations:** 1 Department of Nuclear Medicine, Shanghai Ninth People’s Hospital, Shanghai Jiao Tong University School of Medicine, Shanghai, China; 2 Department of Radiology, Shanghai Ninth People’s Hospital, Shanghai Jiao Tong University School of Medicine, Shanghai, China; 3 Department of Otorhinolaryngology Head and Neck Surgery, Shanghai Children’s Hospital, School of medicine, Shanghai Jiao Tong University, Shanghai, China

**Keywords:** Tomography (X-ray computed), Magnetic resonance imaging, Multimodal imaging, Image-guided biopsy, Head and neck neoplasms

## Abstract

**Objectives:**

To establish an intraoperative CT/MR fusion imaging (FI)-guided core needle biopsy (CNB) approach for head and neck lesions and to evaluate its diagnostic performance in comparison with conventional CT-guided biopsy.

**Material and methods:**

This retrospective study included patients undergoing CT-guided or FI-guided CNB for head and neck lesions between January 2020 and December 2024. Clinical information was collected, including demographics, lesion size, needle insertion depth, number of biopsy cores obtained, and complications. Diagnostic performance was evaluated at both the case-based and core-based levels by comparing biopsy histopathology with the final diagnosis. Subgroup analyses were performed according to lesion size, needle insertion depth, and pathological type.

**Results:**

A total of 152 patients with 426 biopsy cores were included, comprising 66 patients (183 cores) in the CT-guided group and 86 patients (243 cores) in the FI-guided group. At the case-based level, FI-guided biopsy demonstrated significantly higher diagnostic accuracy than CT-guided biopsy (98.8% vs 90.9%, *p* = 0.04), particularly for lesions < 3.7 cm and non-squamous cell carcinoma (non-SCC) lesions. At the core-based level, FI-guided biopsy also achieved higher accuracy (96.3% vs 73.2%, *p* < 0.001) and sensitivity (93.8% vs 65.0%, *p* < 0.001), with consistent advantages across all predefined subgroups. No major procedure-related complications were observed.

**Conclusions:**

FI-guided CNB is a safe and feasible technique for head and neck lesions that improves diagnostic accuracy compared with conventional CT guidance.

**Key Points:**

**Question** Can CT/MR FI overcome the limitations of conventional CT guidance for precise targeting and accurate biopsy of anatomically complex head and neck lesions?

**Findings** CT/MR FI-guided biopsy demonstrated higher diagnostic accuracy at both case-based and core-based analyses than conventional CT-guided biopsy for complex head and neck lesions.

**Critical relevance statement** This study provides evidence that CT/MR fusion-guided biopsy improves targeting precision and diagnostic performance compared with conventional CT-guided biopsy, supporting its application for complex head and neck lesions in clinical radiology practice.

**Graphical Abstract:**

**Figure:**
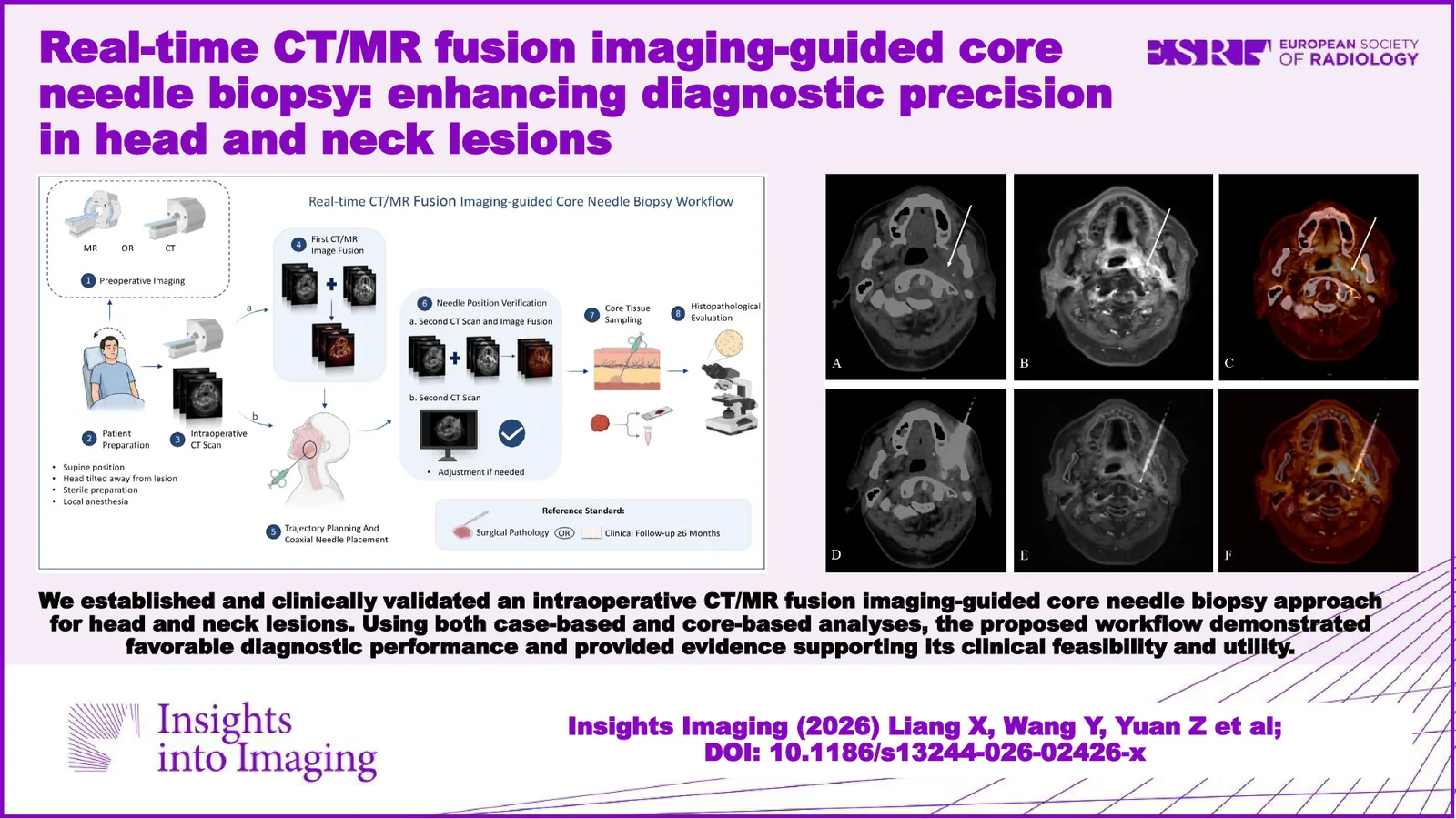

## Introduction

Head and neck cancer (HNC) is the sixth most common type of cancer worldwide and comprises a diverse group of tumors affecting the upper aerodigestive tract [1]. Approximately 5% of annual cancer-related deaths are due to HNC, making it the fifth leading cause of cancer mortality worldwide [2]. Accurate tissue diagnosis is essential for treatment planning, including surgical and nonsurgical strategies such as chemoradiotherapy.

Histopathology remains the gold standard for diagnosis of head and neck tumors (HNTs) [3]. Although open biopsy is the reference standard for tissue sampling, image-guided percutaneous biopsy is increasingly preferred due to its less invasive nature, particularly for nasopharyngeal carcinoma, oropharyngeal squamous cell carcinoma, advanced-stage disease, and deep cervical lymphadenopathy, entities in which upfront surgery is not standard [3, 4]. Furthermore, the anatomy of the head and neck area is complicated due to the confluence of numerous critical structures [5]. This region contains the carotid sheath, parapharyngeal, masticator spaces, pterygopalatine fossa, and is adjacent to critical structures such as the internal carotid artery and facial nerve. Deep-seated lesions are clinically challenging to access, since the complex anatomical structures frequently obstruct direct access to the lesions [6, 7].

Current guidelines for tissue sampling in HNTs recommend percutaneous needle biopsy (PNB), including fine needle aspiration (FNA) or core needle biopsy (CNB) [8]. PNB is minimally invasive and confers a lower risk profile than open biopsies, which is beneficial for elderly patients, individuals with advanced-stage malignancies, and those with significant comorbidities [9, 10]. Among PNB techniques, CNB enables the acquisition of larger tissue specimens while preserving histological architecture, and facilitating more comprehensive pathological evaluation and detailed tumor characterization [11, 12]. Therefore, CNB has become an indispensable diagnostic tool for head and neck lesions, especially when sufficient tissue sampling is required.

Accurate needle placement is crucial to the success of CNB, particularly for deep-seated head and neck lesions; hence, image guidance is routinely employed to ensure safe and precise tissue acquisition. The primary imaging modalities for guiding head and neck biopsies include ultrasound, CT, and magnetic resonance imaging (MRI). Although ultrasound is generally preferred for superficial lesions because of its real-time capability and lack of radiation exposure, its utility for deep-seated targets is often limited by insufficient visualization due to overlying osseous structures or air-containing spaces [13–15]. Computed tomography (CT) has been widely adopted for biopsies of nonpalpable or deeply located lesions, owing to its high spatial resolution and rapid image acquisition [16]. However, our previous study [17] identified poorly defined margins as an independent risk factor for diagnostic failure of CT-guided CNB in deep head and neck lesions. These findings highlight the inherent limitations of CT guidance, including restricted scan-plane orientation and suboptimal soft-tissue contrast [18], and suggest that ill-defined margins or inconspicuous lesions may warrant alternative imaging with superior soft-tissue characterization.

MRI provides superior soft-tissue contrast and more precise delineation of tumor margins and intratumoral heterogeneity, thereby addressing the soft-tissue limitations of CT-guided biopsy [19, 20]. Nevertheless, the routine use of MRI for real-time interventional guidance has been constrained by its stringent magnetic environment requirements, relatively long acquisition time, and procedural complexity [16, 20]. To overcome the complementary limitations of these two modalities, we developed an intraoperative CT/MR fusion imaging (FI)-guided biopsy system. Through image registration, preoperative MR images can be matched with intraoperative CT images, enabling improved visualization of enhancing soft-tissue components and potentially viable tumor regions in multiple planes [21–23]. Given the marked histopathologic heterogeneity of HNTs [24], this approach may facilitate more accurate targeting of diagnostically representative areas during biopsy sampling.

In this study, we present the procedural workflow of this intraoperative CT/MR FI-guided biopsy technique for deep-seated head and neck lesions and evaluate its diagnostic performance in comparison with conventional CT-guided biopsy.

## Material and methods

### Patient population

**Fig. 1:**
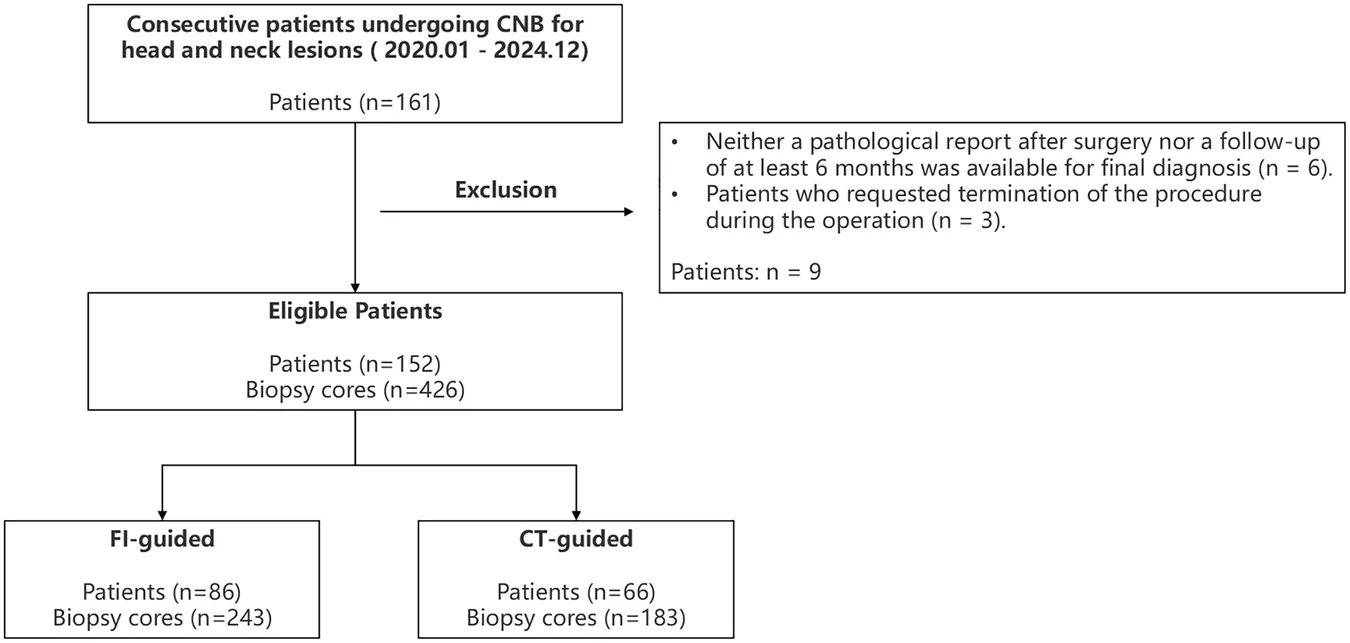
Flow diagram of patient enrollment

This single-center retrospective observational study was approved by the Ethics Review Board of Shanghai Ninth People’s Hospital (IRB approval number: SH9H-2024-TK764-1), and the requirement for written informed consent was waived. Consecutive patients who underwent CT-guided or FI-guided CNB of head and neck lesions for pathological diagnosis at Shanghai Ninth People’s Hospital between January 2020 and December 2024 were included. Biopsy was indicated when pathological sampling was clinically necessary for: (1) initial pathological diagnosis of deep-seated or anatomically complex head and neck lesions; (2) pathological verification of suspected recurrence or residual tumor based on follow-up imaging or clinical assessment after treatment. Residual disease was defined as a persistent abnormality detected within 6 months after treatment completion, whereas locoregional recurrence was defined as a new or enlarging mass or lymph node with contrast enhancement, occurring more than 6 months after treatment, located at or within 3 cm of the primary site [25]. Exclusion criteria were as follows: (1) Neither a pathological report after surgery nor a follow-up of at least 6 months was available for final diagnosis; (2) Patients who requested termination of the biopsy procedure. Patients with contraindications to percutaneous biopsy (e.g., uncorrectable coagulopathy or inability to tolerate the procedure) were not referred for biopsy and therefore were not included in this cohort. The inclusion and exclusion process is detailed in Fig. 1.

For each patient, clinical and radiological data were collected, including age, sex, history of head and neck surgery, lesion location, size, and needle insertion depth (defined as the linear distance from the skin entry point to the needle tip along the needle trajectory), number of biopsy cores acquired, and initial pathological report from the biopsy. The final diagnosis was established either by histopathological confirmation from surgical excision or by clinical–radiological assessment following at least six months of follow-up, based on lesion stability (e.g., no significant size change on serial imaging according to RECIST criteria), progression, or response to treatment (e.g., radiotherapy or chemotherapy). Final diagnoses were established independently of the biopsy results and served as the reference standard for diagnostic performance analysis.

Before June 2022, conventional CT-guided biopsy was the standard biopsy technique for head and neck lesions at our institution. After the introduction of the intraoperative CT/MR FI-guided biopsy workflow in June 2022, FI-guided biopsy was adopted as the routine approach for all eligible patients. Consequently, patients were grouped according to the imaging guidance technique used during the corresponding study period. This sequential implementation reflects routine clinical practice and the institutional transition from conventional CT guidance to FI guidance. To mitigate potential bias arising from the non-randomized design, baseline characteristics were compared between groups and predefined subgroup analyses were performed.

In the FI-guided biopsy group, MRI datasets used for image fusion were derived from pre-existing diagnostic examinations and were required to be of sufficient quality (complete sequences without severe motion artifacts) and to have been acquired within one week prior to biopsy to ensure anatomical comparability. For exploratory analyses, patients were stratified according to lesion long-axis diameter, needle insertion depth, and pathological diagnosis.

### Imaging protocol

Preoperative MR images and intraoperative CT images were acquired according to institutional protocols. CE-T1WI/FS was selected for image fusion because of its superior lesion conspicuity and delineation of tumor margins, particularly for lesions inconspicuous on CT. Detailed acquisition parameters are provided in the Supplementary Text S1.

### Biopsy procedure

All biopsies were performed by the same experienced interventional radiologist with more than five years of experience in head and neck interventions, following a standardized institutional protocol [16].

#### Preoperative imaging

Preprocedural imaging data obtained during routine clinical care were used for lesion assessment and biopsy planning. For FI-guided biopsy, contrast-enhanced MR images acquired within one week before the procedure were used for CT/MR fusion guidance. For CT-guided biopsy, preprocedural MR images could be reviewed during biopsy planning; however, needle targeting and procedural guidance were performed using CT imaging alone without image fusion.

#### Intraoperative CT scan, image fusion, and Biopsy Procedure

Patients maintained a stable supine position with their heads tilted away from the target lesion. The first intraoperative CT scan was performed. For FI-guided biopsy, intraoperative CT images were exported, along with preoperative MR images, into a workstation (United Imaging Healthcare). Image fusion was performed using validated software (LIFEX, version 7.7.0; https://www.lifexsoft.org [26]), which aligned the CT and MRI datasets via rigid registration algorithms. After initial CT/MR fusion, the first registration error was assessed before needle insertion by measuring the maximum spatial discrepancy between corresponding osseous landmarks (defined as the same bony anatomical structures identified on both CT and MR images) adjacent to the target lesion on the fused images. The fused images were subsequently used for biopsy target selection and trajectory planning while avoiding critical neurovascular structures. In CT-guided biopsy, procedures relied solely on intraoperative CT imaging for target localization and needle trajectory determination.

The skin was prepared under sterile conditions using standard disinfection and draping. Local anesthetic infiltration with 2% lidocaine was administered. Utilizing a coaxial biopsy system, a 15-gauge introducer needle (length: 50/110 mm; Demedite Medical Technology) was percutaneously advanced to the lesion under imaging guidance. A second CT scan was then performed after needle placement to confirm the needle position. For FI-guided biopsy, the preoperative MR images were co-registered with the second intraoperative CT images after needle placement, and the second registration error was assessed using the same landmark-based method to confirm needle localization. If the puncture site deviated from the planned trajectory, the coaxial needle was repositioned. For CT-guided biopsy, if the needle placement was deemed inaccurate, adjustments were made under real-time CT guidance until optimal positioning was achieved. Once optimal positioning was confirmed, a 16-gauge core biopsy needle (length: 100/160 mm; Demedite Medical Technology) was then advanced through the introducer needle to obtain tissue samples [27].

#### Sample collection and post-procedure care

**Fig. 2:**
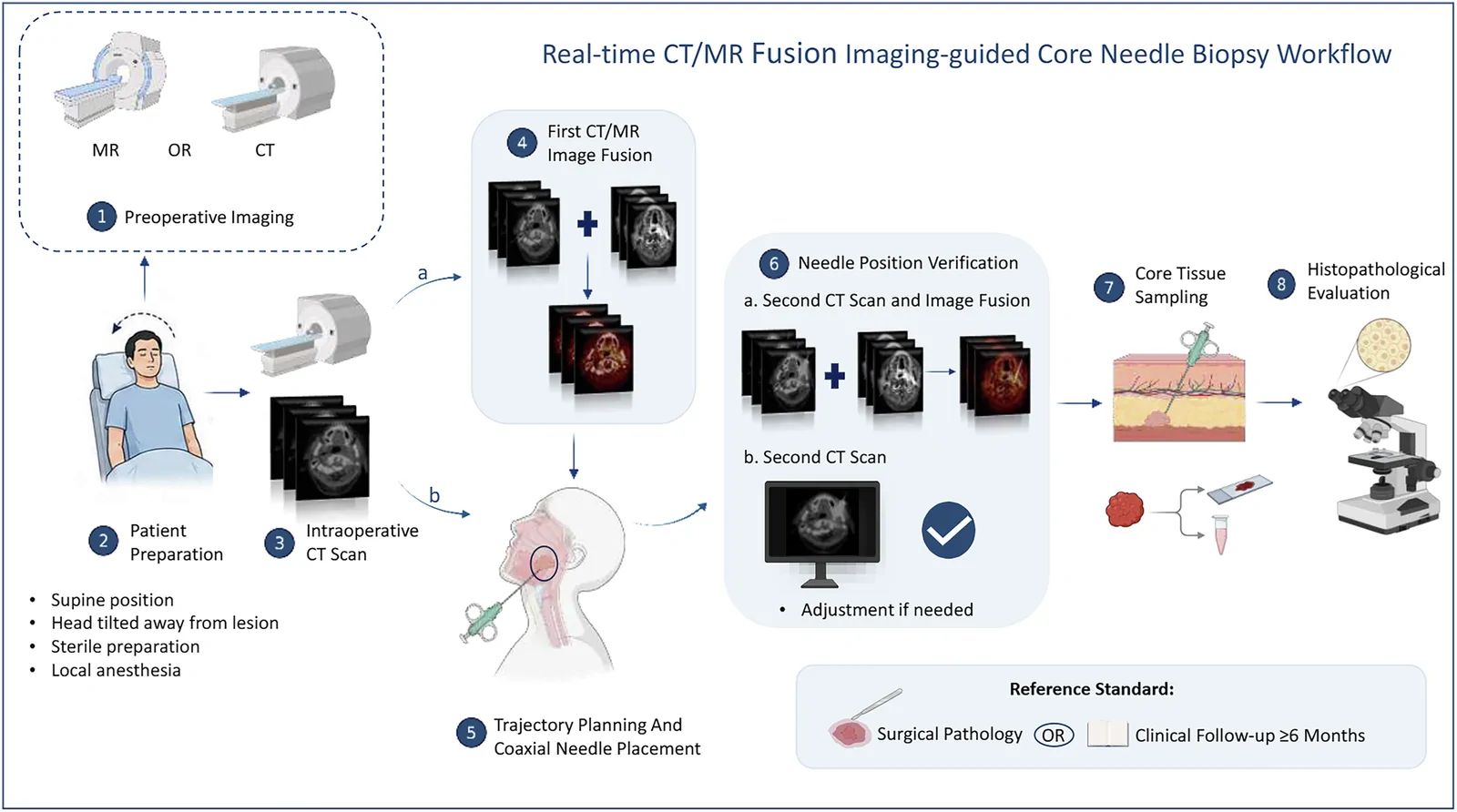
Schematic flowchart: sequential steps of CT-guided or FI-guided head and neck biopsy

The number of biopsy cores was determined based on lesion size, anatomical location, and specimen integrity [6]. All specimens were immediately immersed in 10% neutral buffered formalin and transported to the histopathology laboratory for histologic analysis. Post-procedural hemostasis was achieved through manual compression (duration: 10–15 min) at the puncture site using sterile gauze. To evaluate for acute procedure-related hemorrhage, a limited CT scan was performed immediately following biopsy. If no complications occurred, patients were discharged after 2 h of observation. The sequential steps are summarized in the flowchart (Fig. 2).

### Histopathological evaluation

Initial pathological results were obtained based on acquired biopsy specimens. The biopsy-derived pathological results were determined as correct or incorrect with reference to the final diagnosis for each patient. For patients who underwent surgical excision, histopathological results from surgical specimens were considered the reference standard. For cases without surgical confirmation, the final diagnosis was established based on comprehensive clinical and radiological assessment during follow-up of at least 6 months, according to previously described diagnostic accuracy study methodologies [28–30]. Follow-up assessments were based on serial clinical and radiological evaluations, including evidence of disease stability, lesion progression, or response to treatment. Biopsy cores were classified as accurate if their pathological findings were consistent with the final diagnosis; otherwise, they were deemed inaccurate.

### Statistical analysis

Statistical analyses were performed using SPSS 27.0 (SPSS). A *p* value < 0.05 was considered statistically significant. Kolmogorov–Smirnov (K–S) test was used to assess the normality of the data. Data were presented as means (± standard deviation) in the case of normal distribution or as medians (interquartile range [IQR]) for non-normally distributed variables. Continuous variables were analyzed using the Wilcoxon rank-sum test, Mann–Whitney test, and Student *t*-test according to the distribution normality. We analyzed categorical variables using the χ^2^ test or Fisher's exact test. The diagnostic performance of CT-guided and FI-guided biopsies was assessed with sensitivity, specificity, positive predictive value, negative predictive value, and accuracy, upon case-based and biopsy core-based analyses, respectively. For subgroup analyses, lesion long-axis diameter and needle insertion depth were dichotomized according to the median values of these variables in the study cohort.

## Results

### Patients’ characteristics

A total of 152 cases with 426 biopsy cores were evaluated, including 53.9% (82/152) male and 46.1% (70/152) female patients. The mean age of the patients was 51.5 ± 17.3 years (range: 6–87). A history of head and neck surgery was documented in 38.2% (58/152) of patients. The median (IQR) values for long-axis diameter, short-axis diameter, and needle insertion depth of the lesions were 3.7 cm (IQR: 2.8–5.2 cm), 2.7 cm (IQR: 1.9–3.6 cm), and 3.5 cm (IQR: 2.6–4.6 cm), respectively. The median number of biopsy cores per case was 3 (range, 1–4). The median values of lesion long-axis diameter and needle insertion depth were subsequently used as thresholds for subgroup analyses. Clinical characteristics did not significantly differ between the CT-guided and FI-guided biopsy groups, as detailed in Table 1.

**Table 1:** Baseline characteristics of the patients

Characteristics	Overall	CT-guided	FI-guided	*p* value
No. of patients	152	66	86	
No. of biopsy cores	426	183	243	0.43
Mean age^*^	51.5 ± 17.3	51.9 ± 15.9	50.1 ± 19.3	0.91
Sex				0.07
Female	70	36	34	
Male	82	30	52	
Lesion size (cm)^*^	3.7 (2.8–5.2)	4.0 (2.5–5.3)	3.7 (2.8–4.9)	0.89
< 3.7	2.8 (2.0–3.3)	2.5 (1.8–3.2)	2.8 (2.2–3.3)	0.16
≥ 3.7	5.1 (4.1–5.9)	5.2 (4.3–5.7)	4.8 (4.0–6.3)	0.53
Needle insertion depth (cm)^*^	3.5 (2.6–4.6)	3.2 (2.3–4.1)	3.8 (2.7–4.7)	0.11
< 3.5	2.6 (1.9–2.9)	2.6 (1.8–2.9)	2.6 (2.0–3.0)	0.98
≥ 3.5	4.5 (3.9–5.4)	4.7 (3.8–5.5)	4.4 (3.9–5.3)	1.0
History of H/N surgery	58	27	31	0.54
Initial biopsy results				0.38
Benign	59	23	36	
Malignant	93	43	50	
Lesion type				0.60
Primary	116	49	67	
Recurrent	36	17	19	
Complications				0.76
Hemorrhage	9	6	3	
Infection	3	2	1	

Final diagnosis was established by surgical pathology in 122 patients and by clinical–radiological follow-up of at least 6 months in 30 patients

*p* values were calculated using the Wilcoxon rank-sum test, Mann–Whitney test, or Student *t*-test for continuous variables and the χ² test or Fisher exact test for categorical variables

^*^ Data are means ± SDs or median (IQR)

Final diagnoses were established by surgical pathology in 122 patients, while the remaining 30 were confirmed through clinical and radiological follow-up of at least 6 months. Among the 30 patients diagnosed by follow-up, 4 had benign lesions, and 26 had malignant lesions. Of 152 patients, 61.2% (93/152) were diagnosed with malignancy. Squamous cell carcinoma was the most common malignancy (43.0%, *n* = 40), followed by mucoepidermoid carcinoma (15.1%, *n* = 14) and adenoid cystic carcinoma (8.6%, *n* = 8). Benign diagnoses accounted for 38.8% (59/152) of cases, predominantly chronic inflammation (22.0%, *n* = 13), schwannoma (10.2%, *n* = 6), and pleomorphic adenoma (10.2%, *n* = 6). The distribution of CNB pathological diagnoses in the CT-guided and FI-guided groups is summarized in Supplementary Table S1. Detailed pathological findings of cases categorized as “others” are provided in Supplementary Table S2. Regarding anatomical distribution, nine biopsy targets were located in cervical lymph nodes. The distribution of biopsy target locations, corresponding major CNB pathological diagnoses, and the numbers of cases and biopsy cores at both case-based and core-based levels are summarized in Supplementary Table S3.

No major complications were encountered. Minor complications occurred in 12 patients (7.9%), including postoperative hemorrhage in 9 patients (5.9%) and localized infection in 3 patients (2.0%). Detailed information on complications and their management is provided in Supplementary Text S2.

The median registration error was 1.5 mm (IQR, 1.0–2.1 mm) for the first fusion and 1.5 mm (IQR, 1.0–1.9 mm) for the second (*p* = 0.05); visual assessment confirmed clinically acceptable anatomical alignment.

### Case-based evaluation of diagnostic accuracy

**Fig. 3:**
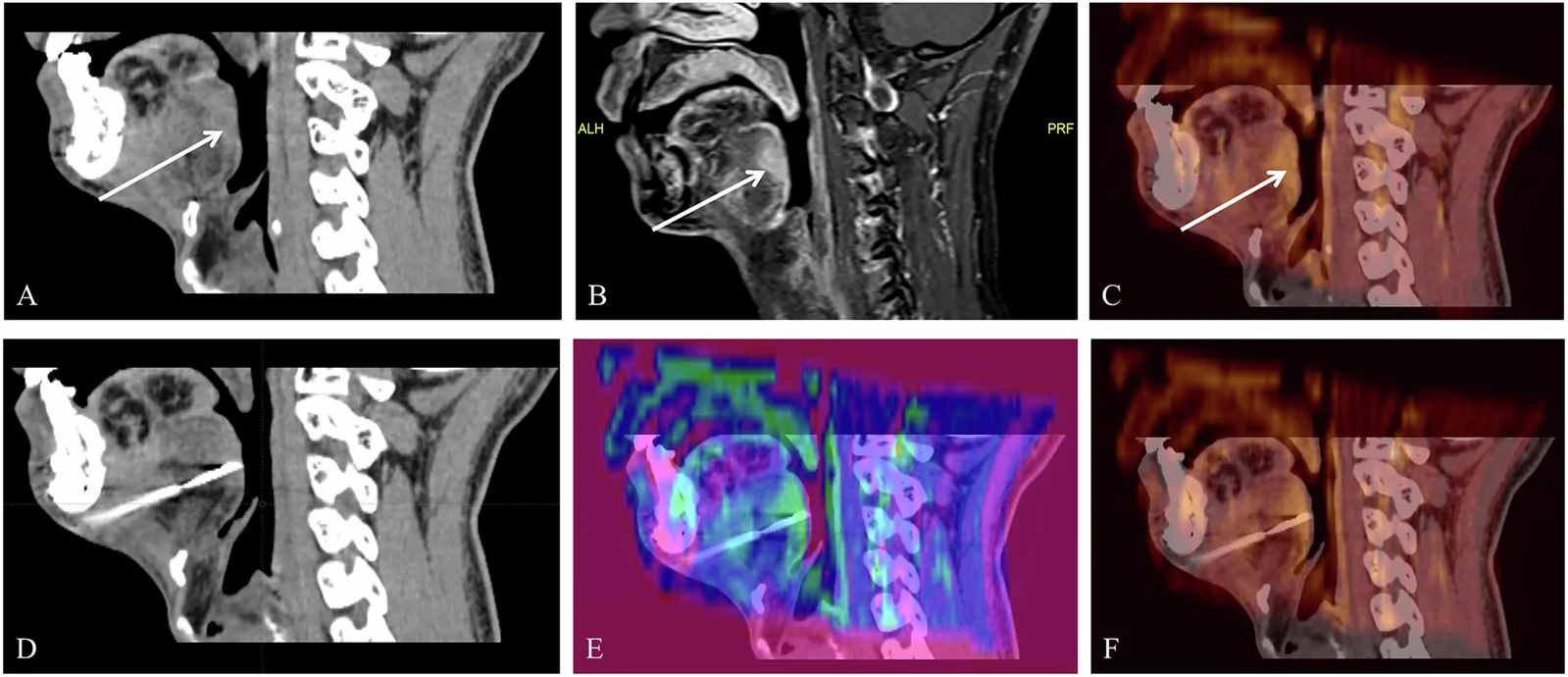
A 42-year-old male patient with a history of squamous cell carcinoma involving the bilateral tongue base presented with restricted mouth opening. Six years later, due to suspected local recurrence, he underwent CNB under FI guidance. Histopathological examination confirmed recurrent squamous cell carcinoma. **A** The first intraoperative sagittal CT scan and (**B**) preoperative contrast-enhanced sagittal T1-weighted MR images show the lesion in the tongue base. **C** Sagittal fusion image before biopsy. **D** Second plain sagittal CT scan after needle placement. **E**, **F** Post-placement fusion images confirm accurate positioning of the biopsy needle within the lesion

**Fig. 4:**
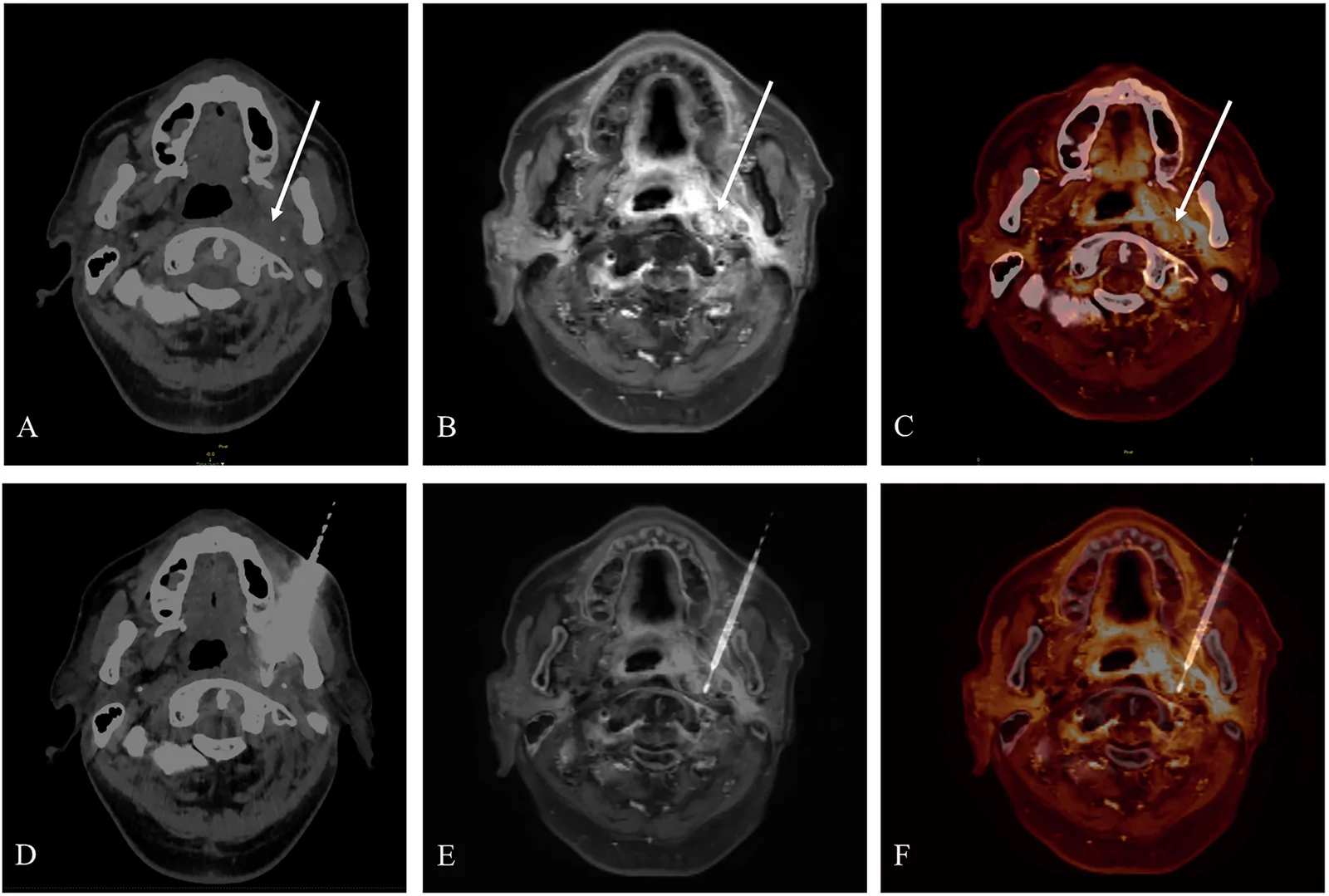
A 62-year-old male patient with a history of nasopharyngeal carcinoma in the left nasopharynx underwent CNB under FI guidance one year later due to suspected local recurrence. Histopathological examination confirmed recurrent nasopharyngeal carcinoma. **A** The first intraoperative axial CT scan and (**B**) preoperative contrast-enhanced axial T1-weighted MR images show the lesion in the left nasopharynx. **C** Axial fusion image prior to biopsy. **D** Second plain axial CT scan after needle placement. **E-F** Post-placement fusion images confirm accurate positioning of the biopsy needle within the lesion

Among 152 patients, the overall diagnostic accuracy was 95.4% (145/152). FI-guided biopsy demonstrated significantly higher accuracy compared to CT guidance (98.8% vs 90.9%, *p* = 0.04), as well as a numerically higher sensitivity (98.0% vs 88.0%), though without statistical significance (*p* = 0.11). In the FI-guided group, one false-negative case was identified. The initial biopsy in this case yielded minimal synovial and cartilage tissue, while final surgical pathology confirmed grade I chondrosarcoma. Before enrollment, 21 patients with high clinical suspicion of malignancy underwent endoscopic biopsy, all of which failed to provide diagnostic pathology. Among the 21 patients, FI-guided biopsy confirmed malignant diagnoses in 20 cases. In the CT-guided group, five lesions initially categorized as benign by biopsy were ultimately confirmed to be malignant through surgical histopathology. Additionally, one CT-guided biopsy failed to sample the target lesion, which was later confirmed to be schwannoma after surgical resection. Representative cases are illustrated in Figs. 3 and 4.

Subgroup analysis revealed that for lesions < 3.7 cm in diameter, FI-guided biopsy achieved significantly higher sensitivity (*p* = 0.04) and diagnostic accuracy (*p* = 0.03) than CT-guided biopsy. In the non-SCC subgroup, FI-guided biopsy also demonstrated higher diagnostic accuracy over CT guidance (*p* = 0.04), with a numerically higher sensitivity that did not reach statistical significance (*p* = 0.10). In other predefined subgroups, FI guidance consistently showed numerically higher sensitivity and accuracy than CT guidance, though these differences were not statistically significant (Table 2).

**Table 2:** Diagnostic values of CT-guided and FI-guided biopsy at case-based level

Subgroup	Diagnostic values	CT-guided	FI-guided	*p* value
Overall (*n* = 152)	Sensitivity	88.0%	98.0%	0.11
	Specificity	100%	100%	-
	PPV	100%	100%	-
	NPV	72.7%	97.3%	-
	Accuracy	90.9%	98.8%	**0.04**
Diameter < 3.7 cm (*n* = 72)	Sensitivity	81.8%	100%	**0.04**
	Specificity	100%	100%	-
	PPV	100%	100%	-
	NPV	66.7%	100%	-
	Accuracy	86.7%	100%	**0.03**
Diameter ≥ 3.7 cm (*n* = 80)	Sensitivity	92.9%	96.0%	1.0
	Specificity	100%	100%	-
	PPV	100%	100%	-
	NPV	80.0%	95.0%	-
	Accuracy	94.4%	97.7%	0.59
Depth < 3.5 cm (*n* = 72)	Sensitivity	82.1%	95.0%	0.38
	Specificity	100%	100%	-
	PPV	100%	100%	-
	NPV	64.3%	93.8%	-
	Accuracy	86.5%	97.1%	0.20
Depth ≥ 3.5 cm (*n* = 80)	Sensitivity	95.5%	100%	0.40
	Specificity	100%	100%	-
	PPV	100%	100%	-
	NPV	87.5%	100%	-
	Accuracy	96.6%	100%	0.36
Non-SCC (*n* = 111)	Sensitivity	82.8%	96.7%	0.1
	Specificity	100%	100%	-
	PPV	100%	100%	-
	NPV	76.2%	97.3%	-
	Accuracy	88.9%	98.5%	**0.04**
SCC (*n* = 41)	Sensitivity	95.2%	100%	1.0
	Specificity	/	/	-
	PPV	100%	100%	-
	NPV	/	/	-
	Accuracy	95.2%	100%	1.0

Specificity and NPV were not estimable (denoted as “/”) because no benign cases were present in the SCC subgroup

*PPV* positive predictive value, *NPV* negative predictive value

Statistically significant *p* < 0.05 values are in bold

### Biopsy core-based diagnostic accuracy

Among the 426 biopsy cores, 353 cores yielded pathological diagnoses concordant with the final diagnosis, resulting in an overall diagnostic accuracy of 82.9%. FI-guided biopsy demonstrated significantly higher sensitivity (93.8% vs 65.0%, *p* < 0.001) and accuracy (96.3% vs 73.2%, *p* < 0.001) compared to CT guidance.

Subgroup analyses further confirmed the superiority of FI guidance across all subgroups. FI-guided biopsy demonstrated significantly higher sensitivity (94.8% vs 46.8%, *p* < 0.001) and accuracy (97.0% vs 59.8%, *p* < 0.001) for lesions with a long-axis diameter < 3.7 cm, while also outperforming CT-guided biopsy in sensitivity (92.5% vs 79.5%, *p* = 0.03) and accuracy (95.5% vs 84.2%, *p* = 0.004) for lesions ≥ 3.7 cm in diameter. When the required needle insertion depth was < 3.5 cm, FI guidance achieved higher sensitivity (90.9% vs 62.8%, *p* < 0.001) and accuracy (94.7% vs 71.3%, *p* < 0.001). Superior performance was also observed when the needle insertion depth was ≥ 3.5 cm (sensitivity: 95.5% vs 67.7%; accuracy: 97.3% vs 75.6%; both *p* < 0.001). Furthermore, FI-guided biopsy consistently demonstrated higher sensitivity and accuracy in both SCC (sensitivity and accuracy: 98.1% vs 63.3%, both *p* < 0.001) and non-SCC subgroups (sensitivity: 91.1% vs 66.3%; accuracy: 95.8% vs 78.1%; both *p* < 0.001) (Table 3).

**Table 3:** Diagnostic values of CT-guided and FI-guided biopsy at core-based level

Subgroup	Diagnostic values	CT-guided	FI-guided	*p* value
Overall (*n* = 426)	Sensitivity	65.0%	93.8%	< 0.001
	Specificity	100%	100%	-
	PPV	100%	100%	-
	NPV	46.7%	91.7%	-
	Accuracy	73.2%	96.3%	< 0.001
Diameter < 3.7 cm (*n* = 197)	Sensitivity	46.8%	94.8%	< 0.001
	Specificity	100%	100%	-
	PPV	100%	100%	-
	NPV	37.7%	93.3%	-
	Accuracy	59.8%	97.0%	< 0.001
Diameter ≥ 3.7 cm (*n* = 229)	Sensitivity	79.5%	92.5%	0.03
	Specificity	100%	100%	-
	PPV	100%	100%	-
	NPV	59.0%	89.6%	-
	Accuracy	84.2%	95.5%	0.004
Depth < 3.5 cm (*n* = 196)	Sensitivity	62.8%	90.9%	< 0.001
	Specificity	100%	100%	-
	PPV	100%	100%	-
	NPV	44.2%	88.9%	-
	Accuracy	71.3%	94.7%	< 0.001
Depth ≥ 3.5 cm (*n* = 230)	Sensitivity	67.7%	95.5%	< 0.001
	Specificity	100%	100%	-
	PPV	100%	100%	-
	NPV	50.0%	93.7%	-
	Accuracy	75.6%	97.3%	< 0.001
Non-SCC (*n* = 312)	Sensitivity	66.3%	91.1%	< 0.001
	Specificity	100%	100%	-
	PPV	100%	100%	-
	NPV	61.4%	92.5%	-
	Accuracy	78.1%	95.8%	< 0.001
SCC (*n* = 114)	Sensitivity	63.3%	98.1%	< 0.001
	Specificity	/	/	-
	PPV	100%	100%	-
	NPV	/	/	-
	Accuracy	63.3%	98.1%	< 0.001

*PPV* positive predictive value, *NPV* negative predictive value

### Variables associated with diagnostic accuracy of biopsy

Univariable analyses were conducted at both the case-based and core-based levels for the following variables: long-axis diameter, needle insertion depth, pathological type, pathological nature, and image-guidance modality. At the case-based level, univariable analysis revealed that only the image-guidance modality (*p* = 0.04) was significantly associated with diagnostic accuracy. At the core-based level, univariable analysis showed that, in addition to the image-guidance modality (*p* < 0.001), needle insertion depth (*p* = 0.02), long-axis diameter (*p* = 0.003) and pathological nature (*p* = 0.01) were also associated with biopsy core accuracy, and were thus included in the multivariable analysis (Table 4). In the multivariable model, lesion long-axis diameter (OR = 1.3; 95% CI: 1.1–1.6; *p* = 0.004) and image-guidance modality (OR = 7.8; 95% CI: 4.1–15.1; *p* < 0.001) remained independent predictors of biopsy core accuracy.

**Table 4:** Univariable and multivariable analyses of factors associated with diagnostic accuracy (case-based and core-based analysis, respectively)

Variable	Univariable analysis		Multivariable Aanalysis	
	OR^*^ (95% CI)	*p* value	OR^*^ (95% CI)	*p* value
Case-based				
Needle insertion depth	1.8 (0.91, 3.43)	0.09		
Lesion long-axis diameter	1.6 (0.92, 2.87)	0.10		
Pathological type, SCC vs non-SCC	2.3 (0.3, 19.6)	0.68		
Pathological nature, benign vs malignant	0.3 (0.04, 2.6)	0.42		
Image-guidance modality, FI-guided vs CT-guided	8.5 (1.0, 72.4)	**0.04**		
Core-based				
Needle insertion depth	1.9 (1.1, 3.1)	**0.02**	0.7 (0.4, 1.3)	0.28
Lesion long-axis diameter	1.3 (1.1, 1.5)	**0.003**	1.3 (1.1, 1.6)	**0.004**
Pathological type, SCC vs non-SCC	0.8 (0.4, 1.3)	0.31		
Pathological nature, benign vs malignant	2.2 (1.18, 3.98)	**0.01**	1.6 (0.8, 3.1)	0.15
Image-guidance modality, FI-guided vs CT-guided	8.6 (4.6, 16.3)	**< 0.001**	7.8 (4.1, 15.1)	**< 0.001**

Only variables identified as significant (*p* < 0.05) in the univariable analyses were entered into the multivariable analysis

^*^ The following reference categories were used: needle insertion depth < median, lesion long‑axis diameter < median, non‑SCC (for pathological type), benign lesions (for pathological nature), and CT‑guided biopsy (for image‑guidance modality). In the core-based analysis, needle insertion depth and lesion long-axis diameter were dichotomized according to their median values

Statistically significant *p* < 0.05 values are in bold

## Discussion

This study established and clinically validated an intraoperative CT/MR FI-guided CNB approach for head and neck lesions. Using both case-based and core-based analyses, the proposed workflow demonstrated favorable diagnostic performance and provided evidence supporting its clinical feasibility and utility. Compared with conventional CT-guided biopsy, FI-guided biopsy achieved significantly higher diagnostic accuracy at the case-based level, particularly in small lesions (≤ 3.7 cm) and non-SCC lesions. At the core level, FI guidance also demonstrated improved accuracy and sensitivity, with consistent performance across all predefined subgroups. Importantly, no major procedure-related complications were observed, supporting the safety and feasibility of this technique in clinical practice. Collectively, these findings indicate that FI-guided biopsy may provide the greatest clinical benefit in scenarios where conventional CT guidance is limited by poor soft-tissue contrast or lesion heterogeneity.

Notably, 20 of 21 patients with prior nondiagnostic endoscopic biopsy results were subsequently diagnosed as malignant tumors using FI-guided biopsy. This improvement in diagnostic yield may be attributable to the reduction of sampling errors associated with subjective visual assessment during endoscopic biopsy, as well as the enhanced ability of FI guidance to precisely target viable solid tumor components. These findings suggest that FI-guided biopsy may be particularly useful in patients with suspected recurrence or prior nondiagnostic biopsy results.

Image fusion has emerged as an important navigation strategy for image-guided interventions by integrating the complementary strengths of different imaging modalities [31]. Fusion of real-time ultrasonography with CT or MRI has been applied for image-guided biopsy and ablation of hepatic lesions by improving lesion conspicuity and target localization; however, its application in the head and neck region is limited by acoustic shadowing from osseous structures and poor visualization of deep-seated lesions, where CT remains the preferred guidance modality [32]. Nevertheless, conventional CT guidance may provide insufficient soft-tissue characterization for delineating tumor extent and viable components. Fusion-based navigation incorporating CT, MRI, and PET has been reported to improve target localization and facilitate biopsy of anatomically complex bone tumors [33], while CT/MR fusion has also facilitated computer-assisted navigation for skull base, temporal bone, and orbital tumors [21, 22, 34]. However, reports on CT/MR fusion-guided CNB of head and neck lesions remain limited. Building upon these previous advances, our study demonstrates the potential utility of CT/MR fusion guidance for biopsy of deep-seated and anatomically complex head and neck lesions.

High-quality tissue acquisition has become increasingly important in modern oncology, particularly with the expanding use of immunohistochemistry, fluorescence in situ hybridization, and next-generation sequencing [35]. These techniques require sufficient and well-preserved tissue specimens, and inadequate sampling may directly affect downstream diagnostic and therapeutic decision-making. Accordingly, we evaluated biopsy performance at both the case and core levels. Our results showed that FI-guided biopsy achieved not only higher diagnostic accuracy at the case-based level, but also improved specimen quality at the core-based level compared with conventional CT-guided biopsy, suggesting a greater likelihood of obtaining diagnostically representative tissue for both histopathologic evaluation and molecular analyses. Notably, while previous studies of head and neck biopsy have mainly relied on case-based analyses, conventional CT-guided biopsy still carries a reported missed diagnosis rate of approximately 10%–27% [10, 11, 36–39]. In the present study, FI-guided biopsy was associated with a substantially lower missed diagnosis rate of approximately 1%–2%.

Additionally, this study addressed a common limitation of conventional image-guided biopsy workflows: the spatial mismatch between preprocedural planning images and intraoperative imaging. Previous technical strategies, such as CT gantry angulation adjustments [6, 40], have been proposed to reduce these discrepancies but may limit procedural flexibility and increase technical complexity [41]. In our workflow, preprocedural MRI was registered with intraoperative CT and displayed in multiplanar reconstruction views, allowing simultaneous assessment of lesion extent and adjacent structures during needle planning. This fusion-guided approach provided more comprehensive spatial information and may contribute to more accurate targeting, particularly in anatomically complex head and neck lesions [42].

The procedural performance of FI-guided biopsy depends on accurate CT/MR image registration. In the present study, both initial and repeated registration procedures demonstrated a median fusion error of 1.5 mm, which is comparable to previously reported CT/MR fusion accuracies [43–45]. Although a marginal difference was observed between the two registration procedures, both achieved clinically acceptable anatomical alignment. However, manual CT/MR registration requires careful selection of stable anatomical landmarks and meticulous verification of image correspondence before needle insertion, particularly in the anatomically complex head and neck region. Therefore, the accuracy and reproducibility of fusion may depend on operator experience and the availability of reliable anatomical landmarks. In our workflow, the interval between the initial intraoperative CT acquisition and the subsequent CT acquisition was approximately 5–10 min, providing a practical reference for the time window required for image fusion. These additional registration procedures may prolong the procedural workflow and increase operational complexity compared with conventional CT-guided biopsy; however, they are essential for achieving reliable fusion guidance. Improving registration consistency through optimized manual workflows or semi-automated fusion algorithms may further enhance targeting precision and reduce operator-dependent variability in clinical practice.

This study has several limitations. First, manual registration is operator-dependent and may introduce variability in fusion accuracy [32]; automated or semi-automated methods could improve consistency. Second, the retrospective, non-randomized design and relatively small sample size may have introduced selection bias, limited the representation of some pathological subtypes and anatomical subsites, and constrained subgroup analyses. Larger prospective, multicenter studies are needed to validate these findings and further evaluate the clinical utility of CT/MR fusion-guided biopsy in head and neck lesions.

## Conclusion

Intraoperative CT/MR FI-guided biopsy is a safe and feasible approach for head and neck lesions that provides higher diagnostic accuracy than conventional CT-guided biopsy. The technique may improve lesion targeting and tissue sampling, particularly in anatomically complex or diagnostically challenging cases.

## Supplementary information

ELECTRONIC SUPPLEMENTARY MATERIAL

## Data Availability

The data that support the findings of this study are not publicly available due to privacy or ethical restrictions, but are available on request from the corresponding author.

## Abbreviations

CNB: Core needle biopsy
CT: Computed tomography
FI: Fusion imaging
HNT: Head and neck tumor
MRI: Magnetic resonance imaging
PNB: Percutaneous needle biopsy
