# Supplementary material for: Real-time CT/MR fusion imaging-guided core needle biopsy: enhancing diagnostic precision in head and neck lesions
Source: Insights Imaging. 2026 Oct 9;17:246. doi: 10.1186/s13244-026-02426-x (PMC13653010; doi:10.1186/s13244-026-02426-x)
Supplement: Supplementary file 1 [file 13244_2026_2426_MOESM1_ESM.pdf]

Enhancing Diagnostic Precision in Head and Neck Lesions

ELECTRONIC SUPPLEMENTARY MATERIALS

Supplementary Text S1. Imaging Protocol

CT procedures were performed on the same CT scanners (Philips Healthcare, Best, Netherlands). Scanner settings were: 120 kV, 200 mAs; section thickness 1.5 mm; slice interval 0 mm; field of view [FOV] 300～ 400 mm. Non-contrast CT was used for image acquisition. Critical vascular structures were assessed using anatomical landmarks and available prior imaging.

MR imaging acquisitions were performed on a 3.0-T scanner (Ingenia; Philips Healthcare). The fusion protocol employed contrast-enhanced fat-saturated T1-weighted imaging (CE-T1WI/FS) with these parameters: repetition time (TR) 580 ms; echo time (TE) 15 ms; slice thickness 4 mm; slice interval 0.4 mm; FOV 250 × 240 mm; matrix 320 × 256. An extracellular gadolinium-based contrast agent (Gadovist, Bayer Pharma AG) was administered at a weight-adjusted dose of 0.1 mmol/kg, with an injection rate of 2 mL/s. CE-T1WI/FS was selected for image fusion because it provides optimal lesion conspicuity and delineation of tumor margins in the head and neck region, particularly for lesions inconspicuous on CT.

Supplementary Text S2. Complications

No major complications were encountered. Postoperative hemorrhage was observed in 5.9% (9/152) of patients, with a median estimated blood loss volume of 19 mL (range, 5–27 mL). All bleeding events were successfully managed by injecting 5 mL of absorbable tissue sealant through the coaxial needle. Localized infection occurred in 2.0% (3/152) of patients and resolved with empirical antibiotics. No acute neurovascular injuries or delayed complications were observed.

Supplementary Table S1. Distribution of initial CNB pathological diagnoses in the CT-guided and FI-guided groups.

Initial CNB diagnosis	Overall	CT-guided	FI-guided	P value
**Benign**				0.81
Chronic inflammation	13	4	9	
Schwannoma	6	2	4	
Pleomorphic adenoma	6	3	3	
Neurofibroma	5	1	4	
Basal cell adenoma	4	1	3	
IMT	4	1	3	
Warthin tumor	3	1	2	
D-TGCT	3	1	2	
LCH	2	1	1	
IgG4-RD	2	0	2	
Others	11	8	3	
**Malignant**				0.17
Squamous cell carcinoma	40	20	20	
Mucoepidermoid carcinoma	14	8	6	
Adenoid cystic carcinoma	8	3	5	
Osteosarcoma	6	5	1	
CXPA	6	1	5	
Rhabdomyosarcoma	5	3	2	
Lymphoma	4	1	3	
Chondrosarcoma	2	0	2	
Others	8	2	6	

CNB, core needle biopsy; IMT, inflammatory myofibroblastic tumor; D-TGCT, diffuse-type tenosynovial giant-cell tumor; LCH, Langerhans cell histiocytosis; IgG4-RD, IgG4-related disease; CXPA, carcinoma ex pleomorphic adenoma

Among benign lesions categorized as “others”, all cases were confirmed by histopathological examination. Among malignant lesions categorized as “others”, two cases were confirmed by clinical–radiological follow-up, and the remaining cases were confirmed by histopathological examination.

Supplementary Table S2. Detailed initial CNB pathological findings of lesions categorized as “others” in Supplementary Table S1.

Category	CNB pathological findings	No. of cases
**Benign**	Skin and skeletal muscle tissue	2
	Teratoma	1
	Nodular fasciitis	1
	Vascular malformation	1
	Rosai–Dorfman disease	1
	Synovial chondromatosis	1
	Nasopharyngeal angiofibroma	1
	Benign glandular tissue	1
	Benign lipomatous proliferation	1
	Synovial and cartilaginous tissue	1
**Malignant**	Malignant mixed tumor	2
	MPNST	1
	Plasmacytoma	1
	Salivary duct carcinoma	1
	Liposarcoma	1
	Neuroendocrine carcinoma	1
	Synovial sarcoma	1

CNB, core needle biopsy; MPNST, malignant peripheral nerve sheath tumor

The pathological findings listed in this table represent the initial pathological results obtained from CNB and were categorized as “others” in Supplementary Table S1 due to their limited case numbers.

Supplementary Table S3. Distribution of biopsy target locations and corresponding CNB pathological diagnoses at case- and core-based levels in CT-guided and FI-guided groups.

Biopsy Target Location	Major CNB pathological diagnoses [a]	Case-based analysis			Core-based analysis		
		Total	CT-guided	FI-guided	Total	CT-guided	FI-guided
Superficial Soft Tissues	Neurofibroma, Chronic inflammation, Nodular fasciitis	7	0	7	22	0	22
Oral Cavity	SCC, Mucoepidermoid carcinoma	4	2	2	12	6	6
Parotid Space [b]	Mucoepidermoid carcinoma, Pleomorphic adenoma, Warthin tumor, Basal cell adenoma, Adenoid cystic carcinoma, SCC	38	15	23	105	40	65
Submandibular Space	SCC, CXPA, Chronic inflammation	19	10	9	55	28	27
Masticator Space/Infratemporal Fossa	SCC, Schwannoma, Rhabdomyosarcoma	22	11	11	63	32	31
Visceral Space	SCC	6	4	2	16	11	5
Pharyngeal Mucosal Space	Adenoid cystic carcinoma, SCC	22	12	10	62	35	27
Parapharyngeal Space	SCC, Lymphoma	3	0	3	9	0	9
Carotid Space	MPNST, SCC, Vascular malformation	5	2	3	13	6	7
Retropharyngeal Space	SCC	1	0	1	2	0	2
Paraspinal Space	Liposarcoma	1	0	1	3	0	3
Maxillofacial Bone	Osteosarcoma, D-TGCT	19	10	9	52	25	27
Pterygopalatine Fossa	Schwannoma, Rhabdomyosarcoma, SCC	5	0	5	12	0	12
Total		152	66	86	426	183	243

SCC, squamous cell carcinoma; CXPA, carcinoma ex pleomorphic adenoma; MPNST, malignant peripheral nerve sheath tumor; D-TGCT, diffuse-type tenosynovial giant-cell tumor

[a] Major pathological diagnoses were summarized for each anatomical region. Diagnoses occurring in ≥3 cases were listed individually; for regions with fewer pathological entities, all observed diagnoses were reported.

[b] Parotid space lesions comprised tumors arising from the superficial and deep lobes of the parotid gland; 22 lesions were located in the superficial lobe and 16 lesions were located in the deep lobe.
